# Comparing drug safety of hepatitis C therapies using post-market data

**DOI:** 10.1186/s12911-019-0860-6

**Published:** 2019-08-08

**Authors:** Jing Huang, Xinyuan Zhang, Jiayi Tong, Jingcheng Du, Rui Duan, Liu Yang, Jason H. Moore, Cui Tao, Yong Chen

**Affiliations:** 1 Departmant of Biostatistics, Epidemiology and Informatics, Perelman School of Medicine, University of Pennsylvania, Philadelphia, PA USA; 2 School of Biomedical Informatics, University of Texas Health Science Center at Houston, Houston, TX USA; 3 Division of Transplant Medicine, Department of Transplantation, Mayo Clinic, Jacksonville, FL USA

**Keywords:** Adverse drug reaction reporting systems, Electronic medical record, Data mining, Hepatitis C, Regulatory decision support

## Abstract

**Background:**

Hepatitis C affects about 3 % of the world’s population. In the United States, about 3.5 million have chronic hepatitis C, and it is the leading cause of liver cancer and the most common indication for liver transplantation. In the last decades, new advances in therapy have substantially increased the cure rate of hepatitis C to more than 95% with the use of antiviral agents. However, drug safety of the new treatments remains one of the major concerns. Data from the US Food and Drug Administration (FDA) Adverse Event Reporting System (FAERS) and the Electronic Health Record (EHR) systems provide crucial post-market information to evaluate drug safety. Currently, quantitative evidence of drug safety of hepatitis C treatments based on post-market data are still limited, and there is also a lack of a standard statistical procedure to systematically compare drug safety across multiple drugs using FAERS and EHR.

**Method:**

In this study, we presented a statistical procedure to compare the difference in adverse events (AE) across multiple hepatitis C drugs using data from FAERS and EHR, and to assess the consistency of results from two data bases. Through three major steps, including descriptive comparison, testing for difference among groups, and quantification of association, the proposed method can provide a quantitative comparison on safety of multiple drugs. Specifically, we compared drugs that were approved by FDA to treat hepatitis C before 2011versus those approved after 2013. We used spontaneous AE reports submitted between 2004 to 2015 from FAERS data base and medical records between 1999 to 2015 from the Cerner health facts data base to estimate and compare the rate of AE after drug use.

**Result:**

We studied 30 most frequently reported AEs after treatment of hepatitis C, comparing the difference between drugs approved before 2011versus those approved after 2013. Our results showed that there was difference in rate of AE between the two groups of treatment. We reported the AEs that have significant statistical difference, and estimate the difference attributable to variation of age and gender between the two groups of drug users. Our findings are consistent with results in existing literature. Moreover, we compared the results obtained from FAERS data and EHR data, and evaluated the consistency of evidence.

**Conclusion:**

The proposed procedure is a general and standardized pipeline that can be used to compare and visualize drug safety among multiple drugs to support regulatory decision-makings using post-market data. We showed that there was statistically significant difference in AE rates between the new and old therapies for hepatitis C. We showed that both FAERS and EHR contained large information for research of post-market drug safety, but each has its own strength and limitations. Cautions should be taken when combining evidence from the two data resources and there is a need of more sophisticated informatics and statistical tools for evidence synthesis.

## Background

Research on post-market drug safety is critical for daily medical practice, especially for newly approved medications [1–4]. It can help us improve the understanding of medical products and protect patients from possible harms that may not be able to identified in pre-market pharmacovigilance studies. The Food and Drug Administration (FDA) have consistently emphasized the importance of monitoring both pre- and post-market adverse events (AEs) after drug use [2], and many efforts have been devoted to achieving the goal. For the pre-market surveillance, potential adverse events were identified in clinical trials and included in the drug labels. However, such information from pre-market clinical trials are limited due to multiple reasons. First, the number of participants in pre-market clinical trial is relatively small comparing to the entire population. It’s also difficult to recruit a diverse study sample in most trials. Second, the duration of pre-market clinical trials is commonly short, which does not allow the identification of AEs that have long incubation period. Moreover, study population in clinical trials usually cannot cover all the special groups, so the identified AEs may not generalize to minority groups, e.g., children and minor ethnic groups. [2, 4]. Due to these reasons, post-market drug surveillance is important for pharmacovigilance research [5].

Spontaneous reporting systems play an important role in the post-market surveillance. For example, the FDA Adverse Event Reporting System (FAERS), which was established for the purpose of national post-market surveillance at 1969, contains over 6 million voluntary spontaneous reports on AEs between 2004 to 2015, from manufacturers, healthcare professionals, and consumers. The FAERS reports were evaluated by the Center for Drug Evaluation and Research and the Center for Biologics Evaluation and Research, and additional investigations could be triggered when a safety concern is identified in FAERS [6]. Several methods have been proposed to study drug safety using data from FAERS and other post-market spontaneous surveillance programs. For example, the proportional reporting ratios and reporting odds ratios methods evaluate the safety of a certain drug by calculating the proportion ratio or odds ratio of a particular AE to all the other AEs for the drug. The Chi-square test [7] can be used to test the dependence between a particular AE and the drug [8]. Bayesian methods were also proposed. For example, the multi-item gamma Poisson shrinker method [9] uses Empirical Bayes method to test the significance of relative reporting rates of a sets of AEs by assigning a common prior to the pre-defined relative reporting rates. The Bayesian confidence propagation neural network method [10] is similar to the multi-item gamma Poisson shrinker but it uses a full Bayesian methodology.

Another important emerging data sources for post-market drug safety research is data from the Electronic Health Records (EHR) systems. Collected through routine clinical care, EHR data provide extensive information on health outcomes and risk factors for a large and broadly representative patient population that cannot typically be derived from traditional randomized clinical trials. Comparing to FAERS, EHR data contain more detailed disease conditions, medication history, and time-related information on patients population. For example, EHR contain information of the diagnoses and events occurred in each clinical encounter as well as the time stamps associated with them, e.g., date of a diagnosis, start and end dates of a prescription, and the dates of hospitalization, discharge and readmission. Such temporal information can be utilized to investigate time-relevant relations between AE and medications. Additionally, EHR data can potentially provide information on a control group which are not standardized in FAERS reports, e.g., patients in the similar disease condition but do not use the same drug. However, limitations do exist for EHR data in pharmacovigilance research. One major challenge is that EHR data are collected based on routine medical practice not for pharmacovigilance research, so the events are recorded using the International Classification of Disease (ICD) codes rather than standardized terminology for AEs. The data elements in EHR may not ideal for pharmacovigilance research, and there could be limited information on AE after drug use in EHR. In contrast, FAERS are specifically designed to study drug safety, the reports were recorded using Medical Dictionary for Regulatory Activities (MedDRA), which is a standardized terminology for AEs [11].

In order to overcome the limitations of each single database and to combine the strength of multiple databases for drug safety research, several efforts have been made very recently to integrate multiple data sources and combine signals. For example, Li et al. have done research combining data from FAERS with EHR to see whether it can improve the performance of AE detection [12]. Rave et al. proposed a signal-detection method combining data from FAERS and EHR by requiring signals from both sources [13]. However, there is still a lack of standard statistical procedure to systematically compare the difference among multiple drugs for specific AEs for data from both FAERS and EHR. Without a standard procedure, the comparison of results or synthesis of evidence from both data resources could be difficult.

In this paper, we proposed a standardized visualization and testing procedure to systematically compare the difference between different drugs for specific AEs and to provide support for regulatory decision-makings. The method could be used for signal detection and hypothesis generation using FAERS data, and is also applicable to EHR data for evidence generation or validation. Our proposed method is easy to implement and interpretable. We illustrated this approach using data from FAERS and Cerner Health Facts EHR Database to compare two groups of therapies for hepatitis C.

According to the statistical data posted by WHO, there are 130–150 million people who have chronic hepatitis C infection. Those who are infected also have a high risk of developing liver cirrhosis, or even liver cancer. Every year, hepatitis C leads to approximately 700,000 death directly or indirectly, which also cause a large number of health care costs [12]. During the last two decades, there have been remarkable advances in drug development which have transformed hepatitis C from a fatal disease to an infection that can be potentially cured [11–13]. Specifically, milestones were achieved at years 2011 and 2013. In 2011, FDA approved the first direct-acting antiviral agents (DAAs), which, combined with pegylated interferon and ribavirin, can improve the cure rates of hepatitis patients with genotype 1 to 70% [14]. However, the benefits of such a combined treatment were diminished by the fact that a large number of patients, who have historically been ‘interferon ineligible or intolerant’ due to pre-existing health conditions, such as mental illness or autoimmune disease, were not recommended to use the combined treatment. In addition, interferon α was found to be associated with multiple side effects, including myelosuppression (anemia, neutropenia and thrombocytopenia), flu-like symptoms and neuropsychiatric side effects (irritability, depression, anxiety and fatigue). It was also found to lower the seizure threshold and exacerbate immune-mediated diseases, which may increase the risk of other medical conditions. [15–17] In 2013, FDA approved new DAAs, which, without a combined medication, can increase the cure rate up to 80–90% for some patients without suffering from the accompanying side effects of combined treatment [18, 19]. In this study, we systematically compare the safety of drugs approved by 2011 and after 2013 using the post-market FAERS and EHR data.

## Methods

### Data source

We considered two data sources in this study, including FAERS and the Cerner Health Facts EHR database. The details of two data sources are listed below.

### FAERS

Since we are interested in comparing hepatitis C treatments approved before 2011 and after 2013, we extracted FAERS reports submitted from 01/01/2004 to12/31/2015, which contains over 6 million reports. In order to standardize the data for reproducibility of the analysis, we normalized the extracted FAERS reports by removing duplicate records and mapping the drug name to RxNorm following Banda’s work [20, 21].

### Cerner health facts

Cerner Health Facts database is a HIPAA-compliant database that collects longitudinal EHR, mostly in-patient data, from multiple Cerner and non-Cerner participating contributing facilities. The database contains rich clinical records, including diagnosis codes, lab test results, hospital admissions, emergency and ambulatory visits, pharmacy, and registration data, creating a nationally representative sample with high-quality data that are readily available for research [15]. We extracted the medical encounters between 1999 to 2015 in the Cerner Health Fact Database.

### Drugs compared

We consider two types of hepatitis C treatments, namely the old and new treatments. The old drugs include: interferon (intron A), ribavirin (copegus, rebetol, ribasphere), boceprevir (victrelis), telaprevir (incivek), peginterferon alfa 2a (pagasys), and peginterferon alfa 2b (pegintron, sylatron), which were approved by FDA before 2011, (generic name outside the parentheses, brand name inside parentheses). The new drugs include harvoni (ledipasvir 90 mg/sofosbuvir 400 mg), sofosbuvir (sovaldi), simeprevir (olysio),viekira (ombitasvir-paritaprevir-ritonavir and dasabuvir), zepatier (elbasvir and grazoprevir), daklinza (daclatasvir), which were approved after 2013. In FAERS, we extracted AE reports that are related to these drugs. In Cerner Health Facts EHR Database, we extracted medical records of hepatitis C patients who have been prescribed with at least one of these drugs. Specifically, we compare patients who have used the old drugs only versus the patients who have used the new drugs only. Patients who have been treated with both old and new drugs were excluded from the study.

### Data analysis

#### Step I: descriptive comparison and visualization

Both the FAERS reports and Cerner Health Facts EHR data contain a large number of reports or medical encounters. Each report in FAERS or medical records for each individual in Cerner Health Facts EHR Database contain at least one AE, and the total number of AEs contained in each report/encounter is one indication of drug safety. Before zooming into a specific report/patient or a specific AE, we compare the distribution of the total number of AEs per report among the two treatment groups using side-by-side histogram plots. We assume that the number of AEs per report/patient could be an indication of drug safety, and such a plot can provide us with a general idea of the number of events per report/patients.

#### Step II: testing for the difference in AE rates among groups

We compared the reporting rates of each AE across the treatment groups using the chi-squared test. The testing procedure will be conducted for multiple AEs. To control the false discovery rate at significance level 0.05, we used Bonferroni correction to adjust for multiple comparisons in order to control the false discovery rate at 0.05. A *p*-value less than 0.05/*m*, which *m* is the number of AEs examined, indicates that the reporting rates of AEs are statistically significantly different between the treatment groups.

#### Step III: quantification and visualization of effect sizes

The testing procedure in Step II can be viewed as a filtering step for dimension reduction, i.e. to reduce the number of AEs to be studied. On the other hand, a large sample size (the number of FAERS reports or patients in EHR) could allow any small difference to be statistically significant. To further investigate the difference in reporting rate of AEs, quantification and visualization of the effect sizes are necessary. In addition, it’s also important to identify patient-level covariates, e.g., gender and age, that could potentially explain the difference, and quantify the proportion of difference that are explained by such variables. To achieve this goal, we propose to conduct the comparisons using logistic regression models, by regressing the occurrence of an AE on treatment groups with or without adjustment of a set of covariates. Specifically, for each report, the outcome is binary with 1 indicating occurrence of the AE, and risk factors are dummy variables indicating therapies. Effect sizes of the association before and after adjusting covariates are compared and visualized using unadjusted and adjusted odds ratios.

## Results

### Description of study population

#### FAERS

We applied the proposed procedure to the FAERS data. We searched FAERS reports submitted from 2004 to 2015 that are related to the drugs we are interested in. The final FAERS dataset includes 43,120 reports in the old treatment only group and 5307 reports in new treatment only group. In Table 1, we summarized the distribution of gender and age, for reports in FAERS and patient data in Cerner Health Facts, respectively. We found that, in FAERs data, patients in the old treatment group were younger than patients in the new treatment group, with a mean difference of 4.1 years old. We did not observe a significant difference in gender distribution between the two treatment groups.

**Table 1:** Distribution of age and gender between old and new treatment groups in FAERS reports and Cerner Health Facts EHR data

	FAERS		EHR	
	Old	New	Old	New
N	43120	5307	820	4363
Age	53.1 (sd = 10.8)	58.1 (sd = 10.2)	51.8(sd =16.8)	47.3(sd =13.1)
Gender (Male%)	55.5%	56.7%	45.1%	63.4%

#### Cerner health facts

**Fig. 1:**
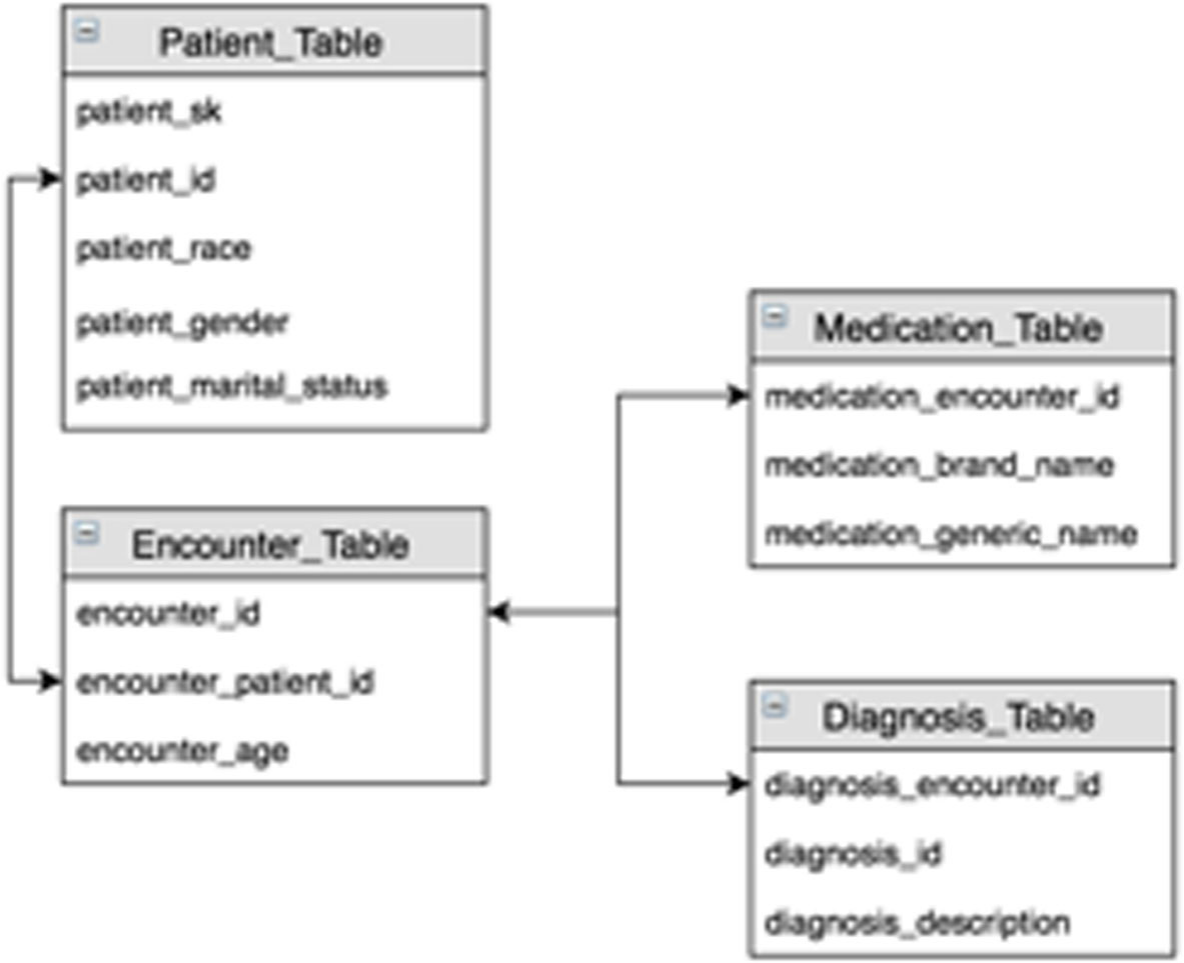
Extraction of medical and demographic information from Cerner Health Facts EHR data

We extracted patients from the Cerner health facts database using the ICD codes for hepatitis C. Patients who had at least one ICD code of hepatitis C, including ICD-9-CM and ICD-10-CM for “Chronic viral hepatitis C”, “Acute hepatitis C”, and “Unspecified viral hepatitis C” were included in the study. The patients were further filtered by using drug names as selection criteria to extract corresponding records from medication table. We used “inner join” for all the tables in Cerner database linked by keys from each table. As shown in Fig. 1, we used arrows to represent the key for joining. For example, we used “patient_id” to link patient table with encounter table, as “patient_id” shared the same information among both tables. We finally extracted 820 patients in the old treatment only group and 4,363 patients in new treatment only group from the Cerner Health Fact Database which contains encounter data from the year of 1999 to 2015. A summary of the distribution of age and gender for the studied subjects are shown in Table 1. In the Cerner EHR data, patients in the old treatment group tend to be older than patients in the new treatment group, with a mean difference of 4.5 years old. The new treatment group tend to have more percentage of males than the old treatment group.

#### Identification and selection of AEs

In FAERS, the AEs are coded using the MedDRA Preferred Terms (PT). In this study, we focused on the top 30 PTs that were mostly reported in the FAERS data among the drug users. However, in the Cerner Health Facts EHR data, the AEs of drugs were not filed specifically. Instead, the state of illness was recorded using International Classification of Diseases (ICD). We mapped the ICD code to MedDRA using UMLS to ensure the analysis using the two different data resources can provide comparable results. For the 30 PTs, 28 of them could be mapped to ICD code using UMLS, and the ICD codes used are listed in Table 2. The other two PTs that we didn’t find any corresponding ICD codes are: “drug ineffective” and “off label use”. Furthermore, we did not find any encounters for AEs fatigue, rash, leukopenia, and blood bilirubin increased among the hepatitis C patients in Cerner EHR. Therefore, we studied 30 PTs using the FAERS, and 24 of the 30 PTs using the Cerner Health Facts EHR Database data.

**Table 2:** ICD codes that are used to identify AEs in the EHR data

PTs	ICD codes	PTs	ICD codes
Fatigue	994.5,994.4	platelet count decreased	287.5
Nausea	787.02	haemoglobin decreased	285.9
Anaemia	285, 285.8, 285.9	weight decreased	783.21
Headache	339.43, 339.8, 339.85, 339.81, 339.09, 339, 339.3, 339.82, 339.4, 339.42, 339.44, 339, 339.83, 339.89, 784	pain	338.1, 338.29, 338.19, 338, 780.96, 338.2, 338.4
Rash	782.1	malaise	780.79, 780.7
Pruritus	698, 698.9, 698.8	arthralgia	719.41, 719.45, 719.4, 719.49, 524.62, 719.4, 719.46, 719.44, 719.43, 719.47, 719.48, 719.42
Vomiting	787, 536.2, 787.03, 787.01	abdominal pain	789.09, 789.07, 789, 789, 789.05
Diarrhoea	564.5, 787.91	thrombocytopenia	287.39, 287.4, 287.5, 287.3, 287.3
Pyrexia	780.6, 780.6, 780.61, 780.63	pneumonia	483, 484.8, 517.1, 997.31, 12.61, 484, 486, 483.8
Asthenia	300.5, 799.3	neutropenia	288.03, 288.09, 288, 288.04, 288
White blood cell count decreased	288.5, 288.59	ascites	568.82, 789.59, 789.5
Dyspnoea	786.05, 786.09, 786	blood bilirubin increased	227.4
Decreased appetite	783	leukopenia	288.5
Dizziness	780.4	pancytopenia	284.1

EHR. Therefore, we studied 30 PTs using the FAERS, and 24 of the 30 PTs using the Cerner Health Facts EHR Database data

### General comparison of distribution of total number of reported AEs per report/patient

**Fig. 2:**
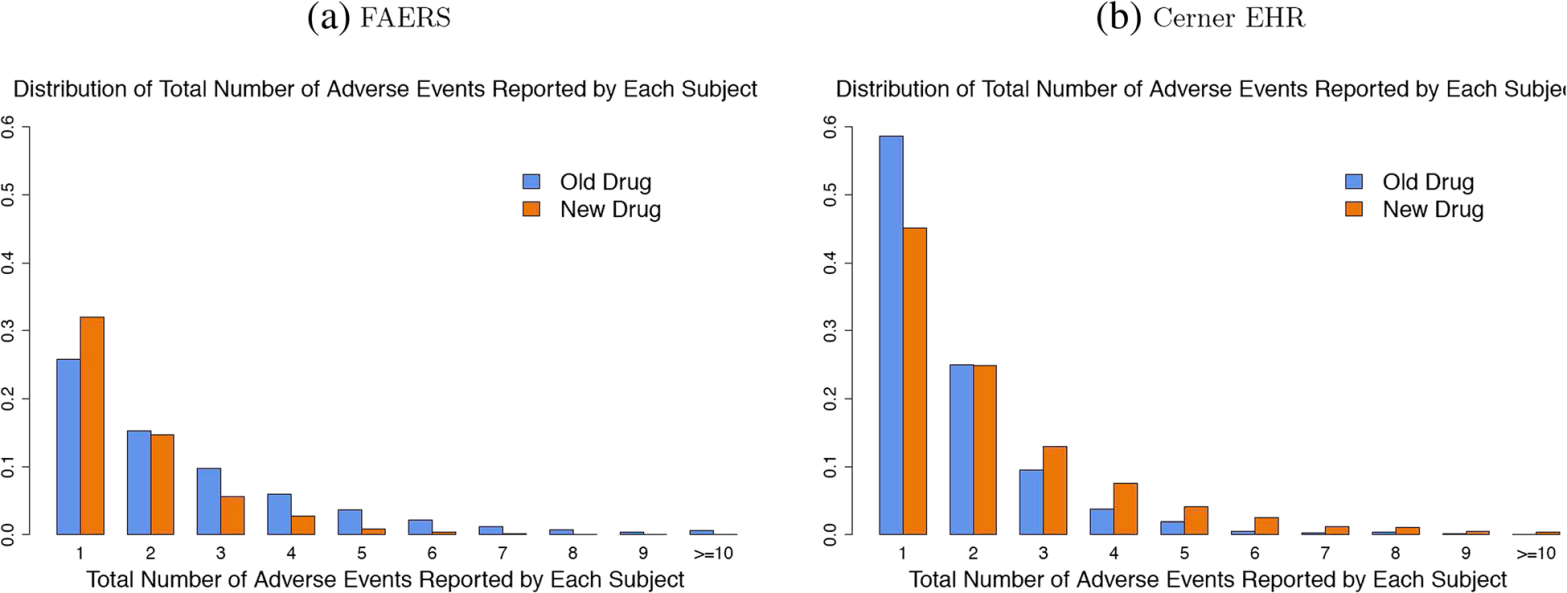
Comparison of distribution of the total number of AEs per FAERS report or patient EHR data in Cerner Health Facts

As the *Step I* of the proposed procedure is to have a descriptive comparison of AE rates between treatment group, we first compared the total number of AEs in each report/patient across the two groups in both FAERS and EHR. Figure 2 showed a histogram plot of the total number of AEs in each report/patient in FAERS and Cerner Health Facts EHR, which visualized the distribution of total number of AEs in each report/patient. A side-by-side comparison of the distribution between the old and new treatment groups were also shown by different colors. Figure 2a demonstrated the results from FAERS and Fig. 2b demonstrated the results from Cerner Health Facts EHR. We found the results were not consistent across the two data bases. In FAERS, it was shown that patients in the new drug group tend to report less number of AEs in each report, which may suggest the old drugs for Hepatitis C may be associated with more AEs than the new drugs, but in the Cerner Health Facts EHR data, we found hepatitis C patients in the new drug group tend to encounter more numbers of AEs.

### Comparison by AE

**Fig. 3:**
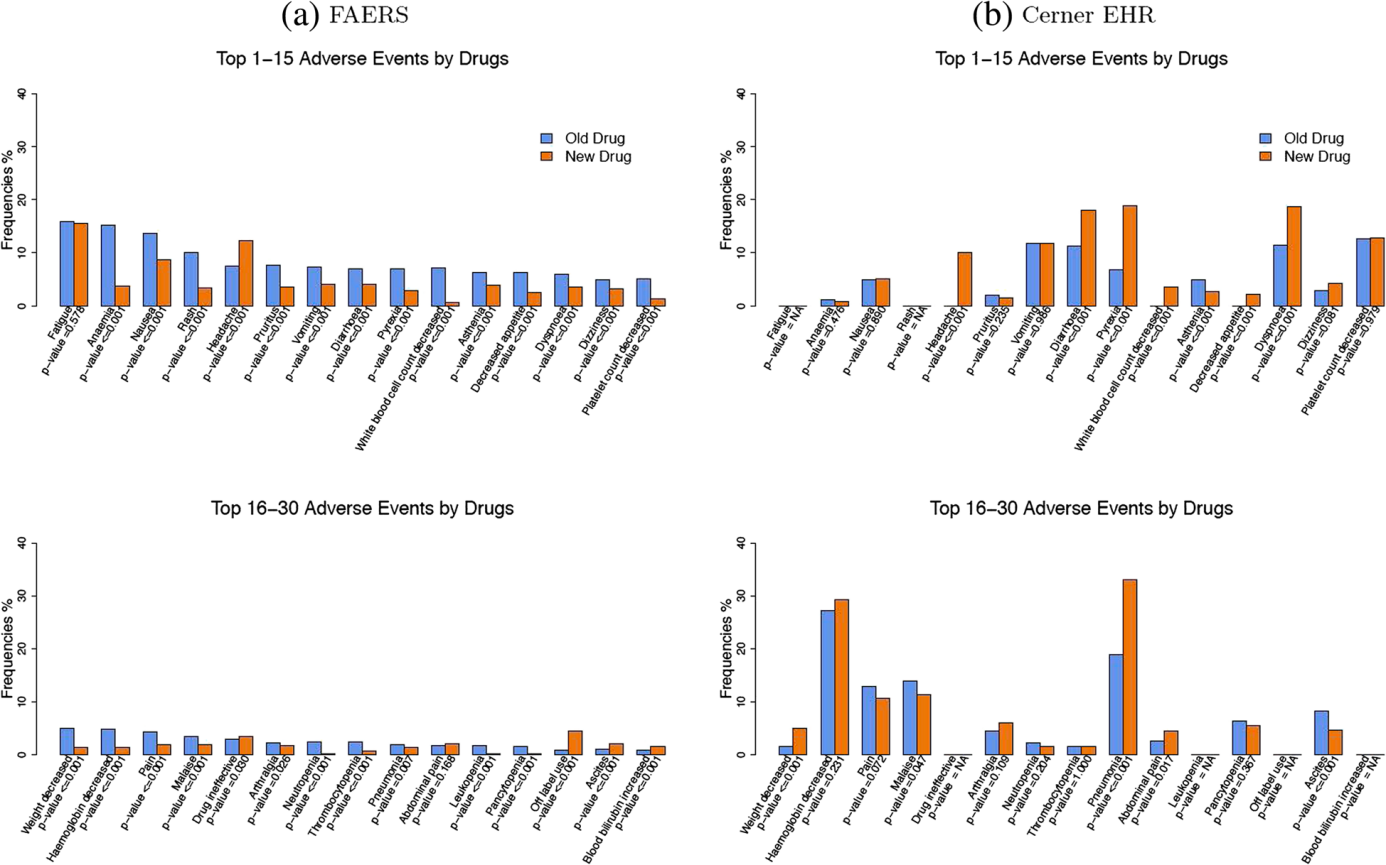
Reporting rates of the top 30 AEs between the two treatment groups

After a descriptive analysis of the total number of AEs per report/patient, in the second step, we compared the rate of AE between two treatment groups for all the 30 AEs. The chi-squared test was used to determine statistical significance of difference, and Bonferroni correction was used to control the false discovery rate. The results were shown in Fig. 3. Specifically, Fig. 3a demonstrated the results from FAERS and Fig. 3b demonstrated the results from Cerner Health Facts EHR data. As shown in Fig. 3a, given the large number of reports in FAERS data, the reporting rate of AEs are significantly different for almost all the 30 AEs, even after Bonferroni correction. Moreover, we found that, in FAERS, patients treated with the old therapies had higher reporting rates in most of the AEs than patients treated with the new therapies, e.g., “anaemia”, “rash” and “pruritus”, while patients with the new therapies only have higher reporting rates in a few AEs than patients with the old therapies, e.g., “fatigue”, “a headache”, “drug ineffective”, “abdominal pain”, “off-label use”, “ascites”, and “blood bilirubin increase.” However, in Cerner Health Facts EHR data, we did not observe significant difference in the reporting rate for most of the 24 AEs. Such a difference in results between the two databases could be due to the fact that FAERS has much larger sample size than Cerner Health Facts EHR. The statistical significance observed in FAERS may be a results of the large sample size, rather than a reflection of any clinically significant difference. Additionally, we found that “pneumomia”, “dyspnoea”, “pyrexia”, “diarrhoea”, “pain”, “weight decreased”, “abdominal pain”, “White blood cell count decreased” were more often seen in patients who used new drugs, while “ascites” and “asthenia” were more frequent in patients who used old drugs in Cerner Health Facts EHR. More importantly, we found the top reported AEs in Cerner Health Facts EHR data is very different from those in FAERS. It may be partially due to the reason that the EHR data could have several medications corresponding to one diagnosis code, which could lead to having mixed AE due to other health conditions.

### Effect of patient characteristics on rate of AE

**Fig. 4:**
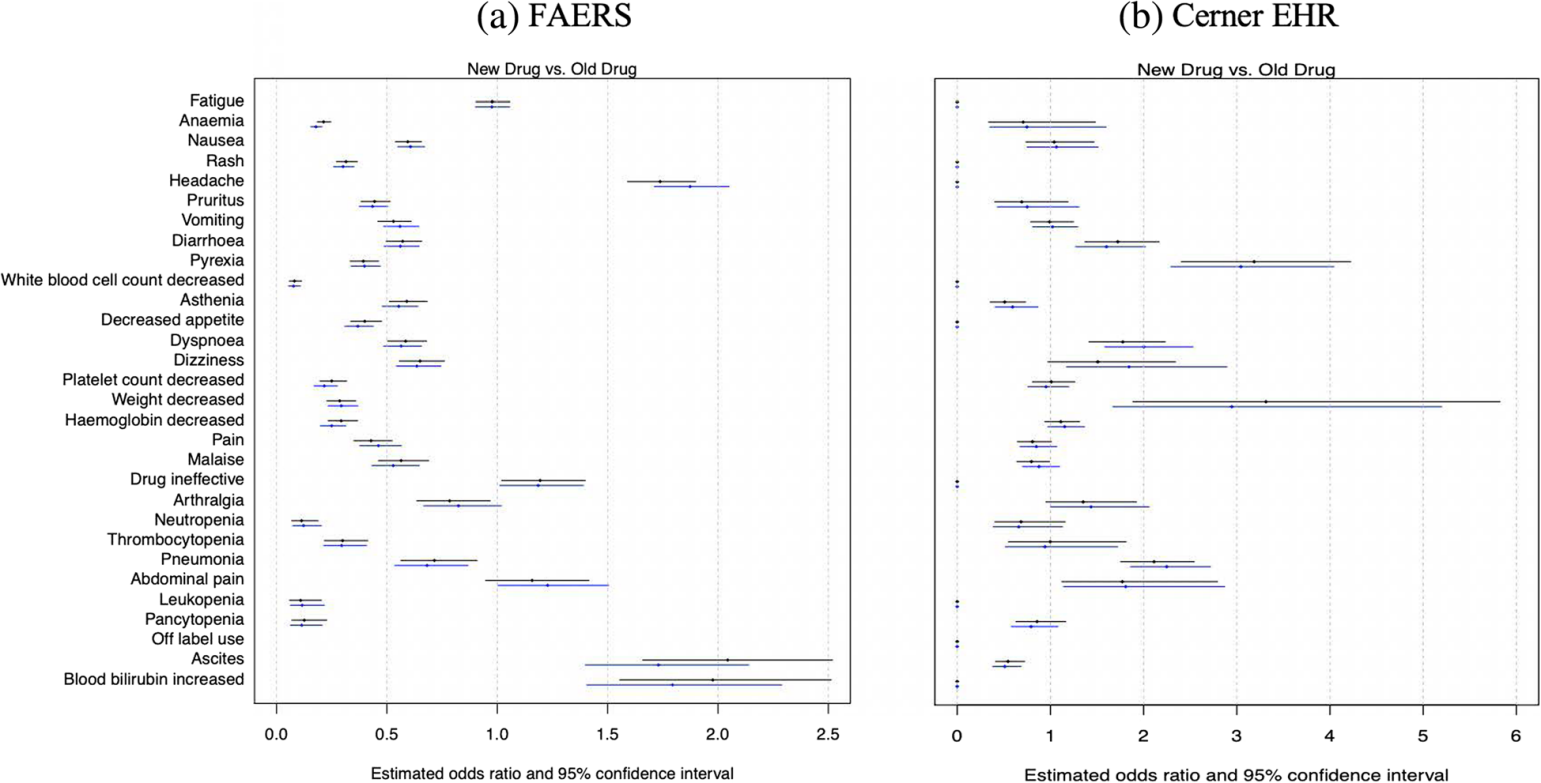
Comparison of estimated odds ratio before and after adjusting for age and gender. The left column compares the new drug group versus the old drug group using the FAERS data, while the right column compares the new drug group versus the old drug group using the Cerner EHR data. The black and blue lines indicate before and after adjusting for age and gender, respectively

As we have mentioned above that the statistical significance observed in FAERS could be due to the large number of reports, rather than true clinically meaning difference, in the third step, we provided effect size estimates to quantify the difference using regression models, and calculate the percentage of difference that could be explained by patient characteristics. Specifically, we contacted logistic regression analyses using the occurrence of a specific AE as a binary outcome (occurred or not) and treatment group as a binary exposure (old versus new therapies). We also consider age and gender as patient-level characteristics to be adjusted in the regression model. Considering the patients with old therapies as the reference group, the regression coefficients of the treatment variable quantified the log odds ratios of experiencing the given AE between patients with new therapies and the patients with old therapies. The magnitude of the regression coefficients is positively related to the strength of association between treatment and AE occurrence. For rare AE, the odds ratio can be used to approximate the relative risk, which directly quantifies the increased (or reduced) risks attributable to the new drugs. Additionally, we evaluated the percentage of increased (or reduced) risk that was attributable to differences in patients characteristics (e.g., difference in age or gender distribution). Specifically, we used the difference between the estimated odds ratios before and after adjusting for patients characteristics to quantify such an attributable risk. The analysis was done for all the 30 AEs. The estimated odds ratios and associated 95% confidence intervals are shown in Fig. 4.

We found that, of the 30 AEs we studied, the odds ratio between the two treatment groups for 12 AEs were decreased after the adjustment, i.e. the adjusted odds ratio is less than the unadjusted odds ratio (attenuate toward 1), and for 18 AEs the adjusted odds ratio was higher than the unadjusted (deviate away from 1) in FAERS data. In the Cerner Health Facts EHR data, the adjusted odds ratio was smaller than the unadjusted for 9 AEs and was higher than the unadjusted for 12 AEs. Overall, age and gender explained the difference in occurrence of AEs between the two treatment groups by 0.2 to 14.8% of the odds ratio.

In Table 3, we show the top 5 AEs that have the largest difference between the adjusted and the unadjusted odds ratios in both FAERS and Cerner Health Facts data bases. In FAERS, the odd of reporting ascites in patients who were treated with old drugs and reported at least one AE is 1.6% (1.5, 1.8%) without adjusting for gender and age and 1.5% (1.4, 1.6%) after adjustment. The odds ratio of reporting ascites between the new therapy and old therapy groups was 2.0 (1.7, 2.5) without adjusting for gender and age and was 1.7(1.4, 2.1) after adjustment. Approximately, 15% of the odds ratio of reporting ascites between the new and old treatment groups could be explained by difference in patient’s gender and age. In Cerner Health Facts HER data, the odds of encountering weight decrease in patients with the new therapies was 3.3(1.9, 5.8) times the odds in patients with the old therapies without accounting for gender and age, and was 3.0(1.7, 5.2) times after adjustment. Difference in patient’s age and gender between the two treatment groups approximately explained 10% of the odds ratio.

**Table 3:** The top 5 AEs with the largest difference between adjusted odds ratio and the unadjusted odds ratio

FAERS			Cerner Health Facts EHR		
AE name	OR(CI) before adjustment	OR(CI) after adjustment	AE name	OR(CI) before adjustment	OR(CI) after adjustment
“Ascites”	2.04 (1.66, 2.52)	1.73 (1.40, 2.14)	“Weight decreased”	3.31(1.88, 5.82)	2.96(1.68, 5.21)
“Blood Bilirubin Increased”	1.98 (1.55, 2.51)	1.79 (1.41, 2.29)	“Dizziness”	1.51(0.97, 2.34)	1.85(1.18, 2.90)
“Headache”	1.74 (1.59, 1.90)	1.87 (1.71, 2.05)	“Dyspnoea”	1.78(1.41, 2.23)	2.01(1.59, 2.54)
“Off Label Use”	5.58 (2.72, 6.58)	5.49 (4.64, 6.51)	“Pneumonia”	2.11(1.75, 2.54)	2.27(1.88, 2.74)
“Abdominal Pain”	1.15 (0.95, 1.42)	1.23 (1.00, 1.51)	“Diarrhoea”	1.72(1.37, 2.17)	1.60(1.27, 2.01)

Comparing the two treatment groups using FAERS reports, patient’s characteristics gender and age could explain more than 10% of the difference in reporting rates for AEs: “anaemia”, “a headache”, “pain”, “ascites”, “platelet count decreased”, “hemoglobin decreased”, and “blood bilirubin increased”. Similar results were also reported in other studies. For example, one study reported that one-third of patients taken interferon reported alopecia, and the rate was higher in females [9].

However, we did not found similar results using the EHR data from Cerner Health Facts, i.e. we applied the same analysis procedure to both FAERS and Cerner Health Facts HER data but the conclusions were consistent. Specifically, the Cerner Health Facts HER data showed that patients treated new drugs encountered more AEs than patient who took the old drugs, which is a different story from the results of FAERS data. It could potentially due to the different data collecting mechanism of FAERS and HER. In FAERS, the data were recorded by report, and multiple report could be associated with the same patients, in Cerner Health Facts HER data, all the encounters of one patient were collected as one record. The Cerner Health Facts EHR data also show the event rates of most of the AEs we studied are more frequent in patients who used the new drugs than used old drugs only.

## Discussion

In this paper, we proposed a standardized statistical procedure that could be used to analyze both FAERS and EHR data for comparison of the difference in rate of AE between different treatment. The proposed procedure provided a standardized testing and visualization tool to evaluate the difference in reporting rates of AEs using post-market data, which were important in pharmacovigilance research and essential to regulatory decision-makings. The proposed three-step procedure including an initial step of descriptive analysis, a second step of dimension reduction and followed by a step of quantification and visualization. It could effectively detect the AEs that have significantly different rates among the therapies, and give insights into how much the difference could be explained by patient’s characteristics.

It was known that both FAERS and EHR data were not perfect for research purpose, but they were large post-market data that contained rich and valuable information for post-market surveillances of drug safety. As a passive reporting system, FAERS data have several limitations. First, the quality of FAERS data is less than optimal. For example, data errors, incompleteness could be common problems in FAERS reports, because reports were self-reported and very few of them were manually validated. As a publicly accessible database, the patient information is also limited in FAERS reports. In particular, the information of patients’ disease conditions was not required in FAERS reports. As the same drug may be used to treat different diseases, patients who take the same drug may have different health condition, and their AEs to the drug could be different. In this situation, the comparison of AEs between two drugs may be unfair. Moreover, patients may take multiple drugs, but FAERS report may not capture that information or could not tell which drug was associated with the reported AE. Additionally, the same patient could report multiple times, with the same AEs for different drugs, and such a behavior may dilute the power of data analysis. Also, in FAERS, very few reports contained time information of the experienced AEs, or potential confounding factors that may affect the association between drug use and occurrence of AEs. In general, FAERS could be used primarily for exploration purpose in pharmacovigilance research, e.g., signal detection, rather than validation or hypothesis testing of any causal relationship. If any safety signal was identified using FAERS data, additional studies need to be conducted to confirm the signal was truly a causal effect.

Several challenges also exist in using the EHR data for pharmacovigilance research. One of the major difficulties is the limited data element and the unique definition of AE in billing codes in EHR. In our investigation of the AEs after Hepatitis C treatments using Cerner Health Facts EHR, we found that the frequency of records for mild AEs are relatively low, which is very difference from FAERS. In addition, the billing code used in EHR was not primarily designed for identification of AEs, which imposed the challenge of mapping the data elements in EHR with other databases that used MedDRA terms to define AEs, e.g., FAERS. Moreover, most of the encounters in the EHR may not be relevant to a specific drug. Additionally, as patients may take multiple medications during the same or overlapped time period, it usually difficult to map the drug with the reported AEs. However, regardless of these challenges, the advantages of EHR data remains. In particularly, the abundant information in EHR, including patients’ medical history, medication and laboratory test results, allows us to design retrospective epidemiological studies to identify patient cohorts are that comparable in health conditions and characteristics, such that the comparison of AEs across treatments could be adjusted for potential confounders. The largest challenge of using EHR data is still the database itself. Since nearly all of the EHR database wasn’t built for post-market surveillance purpose, the adverse reactions are hard to extract. Even though we mapped the ICD code to MedDRA system, the overlapping rate between two terminologists is relatively small.

Additionally, research using either data base will suffer from the challenges of limited and accurate time-stamps of AEs and the lack of the reference patient cohort, i.e., patients who never experience any AE. In FAERS, very few of time-stamps may be found in the narratives of a small percentage of FAERS reports, but it is very difficult to extract them. In EHR data, we may use the date of the corresponding medical record to indicate the time-stamp of an AE, but the accurate AE date is definitely earlier than the record date and the accuracy of such approximation is affected by the schedule of medical visit. Without the temporal information, the comparison of AEs between the two treatment groups could be biased due to differential follow up time periods of new versus existing treatments. For example, in our comparison of new versus old treatments for Hepatitis C, the follow up time of the old drugs is longer than that of the new drug, which is at most 2 years. Then, some long-term AEs of new treatment may not be observed due to the relative short follow up time. Another limitation of our study is the lack of patients who never experiences any AE. Such data is impossible to obtained from FAERS by the nature of passive surveillance system. In EHR data, although we are able to count patients who have zero records of AE, but such a count is also biased by the potential loss to follow up for some patients and differential reporting habits of patients, i.e., some patients may not report minor AEs.

The proposed method can be used as a scanning tool to detect AEs in FAERS and EHR data, thus identifying potential safety signals for further investigations. The hypothesis generated from FAERS or EHR using the proposed procedure could be compared and further studied using other post-market data. The standardized analysis pipeline will increase the comparability of the analysis results using two different data sources. However, in this study, we did not find high consistency in evidence between FAERS and Cerner Health Facts EHR data, and it could be due to multiple factors, including the different data collection mechanisms, different data elements, different terminologies of AEs, and bias sampling in both data bases. As the more and more real-world data are becoming available for pharmacovigilance research, there is still limited guidelines for how to properly conduct the research using these data and limited knowledge about whether such databases are good data resource for pharmacovigilance research. There is a big research gap for standardized methodologies for study design and data analysis in using these real-world data to draw valid conclusion. Our finding revealed a few key challenges in post-market pharmacovigilance research, including the need of a unified terminology system for AEs across different databases, the need of careful study designs for post-market data, and the lack of statistical methods to combine data or evidence from multiple resources that can account for the heterogeneity of patients and data elements across databases. In this paper, we used the 30 most frequent reported AEs in FAERS for illustration of the proposed procedure, but in many situations, rare events, such as death, can be of greater clinical importance and research interest. The proposed method may have low power in analyzing the rare events, as it could potentially suffer from the sparsity of the signal. Extension of the current method to rare events is currently under investigation and will be reported in the future.

In our investigation, we studied the drug safety of new versus old drugs for hepatitis C, which is a relatively common disease condition. We were able to extract a large number of reports and patient from both FAERS and Cerner Health Facts EHR Database, and the analyses were well powered. In other situations, for example, rare disease conditions, the study of drug safety using one single EHR database could be problematic, because a single EHR database may not be able to provide enough patients for the drug safety research. For instance, a drug could be related to a couple of AEs, but given one patient, s/he may experience only a small subset of the AEs. This could lead to biased study results. It is also true for study of rare AEs. In order to use EHR data to study rare AEs or the safety of drugs that are used to treat rare disease condition, it is important to combine or integrate EHR from multiple hospitals or healthcare providers. EHR from different sites of large consortiums, e.g., the Observational Health Data Sciences and Informatics program (OHDSI), and PEDSnet, could be used to empower the pharmacovigilance research of treatments for rare conditions or rare adverse reactions. However, such studies using FAERS may not be affected by the limited number of sample size, because FAERS receives reports from nationwide.

The proposed procedure could also be extended. For example, in the third step: quantification and visualization of effect sizes, we used logistic regression model to estimate the odds ratio of experiencing AEs between two different groups. In order to use such a model, the AE outcome is coded as a binary variable, with 1 indicating the AE occurred and 0 indicating not occurred. However, in reality, a patients may experience the same AE multiple times. In this scenario, the AE outcome could also be coded as number of events for the AE, which includes more information on the drug safety than a binary outcome. In our analysis, we transformed the outcome variable, count of AE, to a binary variable, occurrence of AE. Such a transformation may lose information. In the future, we could use Poisson regression model, in addition to the logistic regression model in Step III to study the rate of AE and rate ratio of AE between two treatment group. Such an extension could also incorporate the zero-inflated feature of the data, e.g., many AEs may never occur in a couple of patients. We are currently working on the extensions and developing an R package, termed as “AEtools”, to semi-automate the proposed pipeline for the statistical analysis and visualization.

## Conclusions

The proposed procedure is a general and standardized pipeline that can be used to compare and visualize drug safety among multiple drugs to support regulatory decision-makings using post-market data. Though our analysis, we found there was statistically significant difference in AE rates between the new and old therapies for hepatitis C, but the conclusions from FAERS and EHR data were not consistent. We showed that both FAERS and EHR contained large information for research of post-market drug safety, but each has its own strength and limitations. Cautions should be taken when combining evidence from the two data resources and there is a need of more sophisticated informatics and statistical tools for evidence synthesis.

## Data Availability

FAERS data can be downloaded from https://fis.fda.gov/extensions/FPD-QDE-FAERS/FPD-QDE-FAERS.html. The deidentified data extracted from Cerner Health Facts and the R code for this project can be accessed from https://github.com/Penncil/Comparing-drug-safety-of-Hepatitis-C-therapies-using-post-market-data.

## Abbreviations

AE: Adverse events
DAAs: Direct-acting antiviral agents
EHR: Electronic Health Record
FAERS: FDA Adverse Event Reporting System
FDA: US Food and Drug Administration
ICD: International Classification of Disease
MedDRA: Medical Dictionary for Regulatory Activities
OHDSI: Observational Health Date Sciences and Informatics program
PEDSnet: Pediatric EHR Data Sharing Network
PT: Preferred Terms
UMLS: Unified Medical Language System
WHO: World Health Organization
